# Reconstruction of Complex Network based on the Noise via QR Decomposition and Compressed Sensing

**DOI:** 10.1038/s41598-017-15181-3

**Published:** 2017-11-08

**Authors:** Lixiang Li, Dafei Xu, Haipeng Peng, Jürgen Kurths, Yixian Yang

**Affiliations:** 1 Information Security Center, State Key Laboratory of Networking and Switching Technology, Beijing University of Posts and Telecommunications, Beijing, 100876 China; 2 National Engineering Laboratory for Disaster Backup and Recovery, Beijing University of Posts and Telecommunications, Beijing, 100876 China; 3 Potsdam Institute for Climate Impact Research, Potsdam, D14473 Germany; 4 State Key Laboratory of Public Big Data, Guizhou, 550025 China

## Abstract

It is generally known that the states of network nodes are stable and have strong correlations in a linear network system. We find that without the control input, the method of compressed sensing can not succeed in reconstructing complex networks in which the states of nodes are generated through the linear network system. However, noise can drive the dynamics between nodes to break the stability of the system state. Therefore, a new method integrating QR decomposition and compressed sensing is proposed to solve the reconstruction problem of complex networks under the assistance of the input noise. The state matrix of the system is decomposed by QR decomposition. We construct the measurement matrix with the aid of Gaussian noise so that the sparse input matrix can be reconstructed by compressed sensing. We also discover that noise can build a bridge between the dynamics and the topological structure. Experiments are presented to show that the proposed method is more accurate and more efficient to reconstruct four model networks and six real networks by the comparisons between the proposed method and only compressed sensing. In addition, the proposed method can reconstruct not only the sparse complex networks, but also the dense complex networks.

## Introduction

Complex networks show a high degree of complexity and they can abstractly describe a large number of real systems in the fields of biology, economy, society, physics and etc. At present, the link prediction^1–3^, the structure^4–7^, the dynamical behavior^8,9^ and etc. are hot research issues in the field of complex network. Especially, the topological structure of networks is crucial for the research on dynamic properties of complex networks. It is very important to study the relationship between the topological structures and various dynamical behaviors of complex networks for understanding and controlling complex network systems. The reconstruction of complex networks is a challenging inverse problem. In general, the network structure and the node dynamics are unknown, and only the time series can be measured. At times like that only limited data can be obtained from the dynamics of individual units of the networks, it is impossible to directly measure the interactions between nodes, which leads that the network topology can not be obtained directly. The reconstruction of complex networks plays an important role in many areas, such as inferring gene regulatory networks from expression data in biological networks^10,11^, predicting information dissemination and virus transmission in social network^12^ and so on.

Many methods were developed to deal with the problem of the network reconstruction, such as Bayesian reasoning^13^, ODE^14^, Lasso^15^ and so on. The linear model has received a lot of attentions in the network reconstruction, because many real systems can be described as linear or approximate linear equations^16^. For a linear system with deterministic inputs, Gonçalves and Warnick^17^ considered that a certain number of inputs were needed to solve the reconstruction problem. Materassi and Innocenti^18^ also mentioned that reliable estimations could be obtained with sufficient data. For linear systems, Chang and Tomlin^19^ used the single-perturbation time series to stimulate all the distinct states, and used a data-driven reasoning method to identify biological networks. The disadvantage of their approaches was that enough data were needed to be observed. How to reduce measurement data to achieve accurate network reconstruction is an important research problem.

Noise is ubiquitous in physical and natural systems, and the data measured from linear systems are inevitably influenced by the noise. However, Madni did not consider the noise in solving the reconstruction problem of sparse networks^20^. The presence of noise may be a double-edged sword. On the one hand, many people believed that the existence of noise was harmful to the network reconstruction. Experimental results of Shen *et al*.^12^ showed that the success rate of reconstruction was reduced by the fraction *n*_*f*_ of states in the time series that flipped due to noise. Minimum data increased for achieving at least 0.95 AUROC (area under the receiver operating characteristic curve) and AUPR (area under the precision-recall curve) simultaneously with the increment of noise^15^. On the other hand, the proper use of noise may be beneficial to the network reconstruction. The existence of noise led to a general, one-to-one correspondence between the dynamical correlation and the connections among the oscillators for a variety of node dynamics and network structures^21^. Thus, the states of network nodes in the linear network system can be changed greatly by the stimulation of noise.

For the network reconstruction with less measurement data, the compressed sensing is an efficient method and it only acquires a smaller amount of sample data to recover the sparse signal. Shen *et al*.^12^ introduced in detail the reconstruction of diverse propagation networks and the identification of hidden sources based on compressed sensing. Wang *et al*.^22^ proposed the reconstruction of complex networks based on the evolutionary game data via compressed sensing. Besides, the reconstruction of dynamical network based on compressed sensing was studied^23,24^. Most of the existing researches studied the sparse networks. However, there are not only sparse networks, but also dense networks. How to efficiently reconstruct the dense networks is an urgent problem to be solved. Currently, the relevant research is still very few.

This paper presents a new method to solve the reconstruction problem of complex networks whose node states are generated by the linear network system. The state of simple linear systems is stable, and it has a strong coherence, which brings difficulty to the reconstruction of complex networks generated by the linear system. Therefore, Gaussian noise is used as the input to make the linear system oscillate and it breaks the stability of the system state. We decompose the state matrix of the linear system by QR decomposition, construct the measurement matrix by Gaussian noise, and reconstruct the input sparse matrix based on compressed sensing. Thus, the structure of complex network can be reconstructed. We discover that the noise can build the bridge between the dynamics and the topological structure in order to realize the network reconstruction. The proposed approach can efficiently reconstruct both the sparse and dense networks. We validate the applicability and the efficiency of the proposed approach for the sparse and dense networks by utilizing four different model networks and several real networks. We discover that only less measurement data are required by the proposed method to reconstruct the network after adding the Gaussian noise, which will increase the success rate of network reconstruction.

## Results

### Network reconstruction without control input

We consider the reconstruction problem of complex networks whose node states are generated by the following linear network system without control input

1 $$\dot{X}(t)=AX(t)$$

where the vector $X(t)={({x}_{1}(t),{x}_{2}(t),\cdots ,{x}_{N}(t))}^{T}$ is the state of *N* nodes for a network system at time *t*, and this $N\times N$-dimensional matrix *A* is the network structure between nodes. From Equation (1), we have

2 $${Y}^{T}={X}^{T}{\bar{A}}^{T}$$

where ${X}^{T}$, ${Y}^{T}$ represent the input and output matrices which are generated by *P* experiments, ${X}^{T}$ is the $P\times N$-dimensional matrix of the system, ${Y}^{T}$ is a $P\times N$-dimensional matrix. For the detailed explanation of ${\bar{A}}^{T}$, please see Eq. (15) in the Methods section.

Now we consider the reconstruction problem of six different real networks and four model networks, namely, a network of books about US politics (Polbooks)^12^, a neural network of the nematode C. Elegans (Celegansneural)^25^, a social network of dolphins (Dolphins)^26^, the network of American football games in the Fall of the year 2000 (Football)^27^, a network of jazz musicians (Jazz)^28^, a social network of friendships of a karate club (ZK)^29^, the Newman-Watts small-world network (NW)^30^, the Watts-Strogatz small-world network (WS)^31^, the Erdos-Renyi random network (ER)^32^, and the Barabasi-Albert scale-free network (BA)^33^. The nodes states of these networks are generated though the linear network system (1). These networks are sparse (the sparsity $k\ll N$), and the average sparsity of each network is shown in Table 1. In Table 1, *N* is the size of the network, *L* is the links number of network nodes, $\langle k\rangle$ is the average sparsity of the network, and *nt* is the ratio between the row and the column of matrix ${X}^{T}$ in Eq. (2), i.e. $nt=P/N{\rm{(0.1}}N\le P\le 4N)$.

**Table 1:** In the linear network system without control input, the reconstruction success rates by three different methods for some networks, i.e. Polbooks, Celegansneural, Dolphins, Football, Jazz, ZK, NW, WS, ER and BA, where *N* is the size of the network, *L* is the links number of network nodes, $\langle k\rangle$ is the average sparsity of the network, *nt* is the ratio between the row and the column of matrix ${X}^{T}$ in Eq. (2), i.e. $nt=P/N$, and $P$ is the number of experiments ($0.1N\le P\le 4N$).

Networks	N	L	$\langle {\boldsymbol{k}}\rangle$	Success rates as ${\bf{0.1}}\le {\boldsymbol{nt}}\le {\bf{4}}$
Polbooks	105	441	8.4	0
Celegansneural	297	2359	14.5	0
Dolphins	62	159	5.1	0
Football	115	613	10.7	0
Jazz	198	5484	27.7	0
ZK	34	78	4.6	0
NW	100	416	4.2	0
WS	100	400	4.0	0
ER	100	406	4.1	0
BA	100	390	3.9	0

Due to the rows of matrix ${X}^{T}$ can be controlled, we consider reconstructing these networks in three different methods according to three cases of *nt*. Table 1 shows the success rate of the reconstruction for each network at $0.1\le nt\le 4$. From Table 1, we can see that

i. When ${X}^{T}$ is an underdetermined matrix (i.e. $0.1\le nt < 1$), we reconstruct these networks by the compressed sensing method. For the detailed explanation of compressed sensing, please see the Methods section. And the success rates of networks reconstruction are 0;
ii. When ${X}^{T}$ is an $N\times N$-dimensional matrix (i.e. $nt=1$), we reconstruct these networks by solving the inverse matrix method. But the reconstruction success rates of these networks are all 0;
iii. When ${X}^{T}$ is an overdetermined matrix (i.e. $1 < nt\le 4$), we reconstruct these networks by the least square method, which is implemented directly in a function provided by MATLAB simulation software. But the success rates of these networks are still 0.

In other words, these networks can not be reconstructed in these three cases of *nt*.

**Figure 1:**
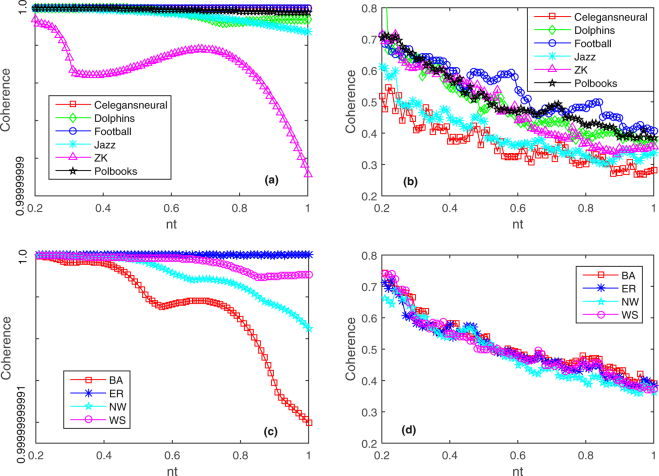
Coherence of measurement matrices as a fraction of *nt*. The networks size *N* is shown in Table 1, *P* is the number of experiments, and we select the measurement data from the time $t=350$. (**a**) When the input vector $u=0$, the coherences of six measurement matrices for these six networks, i.e. Celegansneural, Dolphins, Football, Jazz, ZK, Polbooks vary with the increment of *nt* (now $nt=P/N$, $0.2N\le P\le N$). (**b**) When the input vector *u* is the standard Gaussian noise, the coherences of six measurement matrices for these six real networks change with the increment of *nt* (now $nt=(P-N)/M$, $1.2N\le P\le 2N$, and we fix $M=N$). (**c**) When the input vector $u=0$, the coherence values of measurement matrices for these four networks (i.e. Erdos-Renyi random network (ER), Newman-Watts small-world network (NW), Barabási-Albert scale-free network (BA) and Watts-Strogatz small-world network (WS)) created through the linear network system change with the increment of *nt* (now $nt=P/N$, $0.2N\le P\le N$). (**d**) When the input vector *u* is the standard Gaussian noise, the coherence values of these four measurement matrices for these four model networks change with the increment of *nt* (now $nt=(P-N)/M$, $1.2N\le P\le 2N$, and we fix $M=N$).

We analyze the reasons why these networks can not be reconstructed though the linear network system. When the system has no control input, the state $X(t)$ of the system will eventually present a stable state and have a strong correlation. In order to verify the strong coherence of the state $X(t)$ for the linear network system without control input, we select the measurement data from the time $t=350$, gradually increase *nt* (that is, we increase the experiments number *P*, and the size *N* of the network is fixed) and obtain the coherence changes of the state matrices ${X}^{T}$ generated by these networks. The coherence is calculated according to Eq. (8). In Fig. 1(a, c), the curves with different marks represent the coherence changes of the state matrices for six real networks and four model networks. The values of *N* are shown in Table 1, and the range of *P* is $0.2N\le P\le N$. It can be seen from Fig. 1(a) that the coherence of the state matrix for ZK network presents the volatility, while the coherences of state matrices for the rest five networks appear in a decreasing trend with the increment of *nt*. But in a whole, the coherences of these six state matrices are still close to one (since the coherence of the state matrix is relatively strong when *nt* is too small, the simulation of Fig. 1 starts with $nt=0.2$). The coherence curves of the state matrices for BA, NW and WS networks appear at a decreased trend with the increment of *nt* in Fig. 1(c), where the coherence of the state matrix for BA network changes more greatly, but the coherences of these four model networks are all close to one. These simulation results in Fig. 1(a) and Fig. 1(c) present the stable and strong coherence state of the linear network system without control input. So we can not reconstruct these networks through the linear network system without control input.

### Network reconstruction with control input

**Figure 2:**
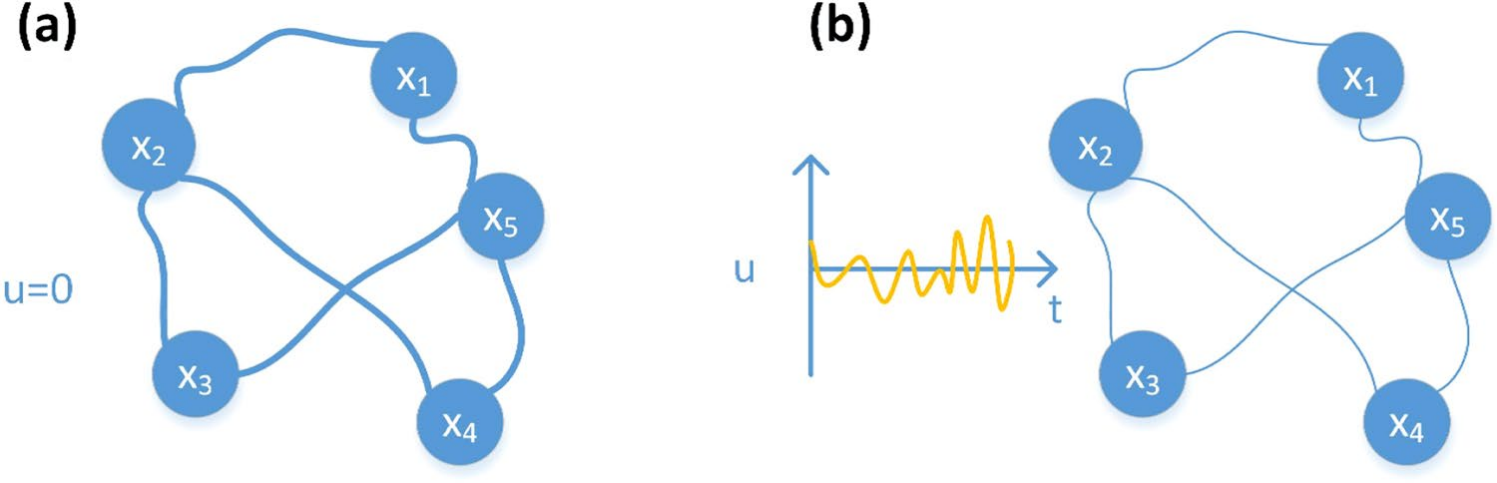
Intensity changes of states coherence for a linear network system. (**a**) When the input vector $u=0$, the coherence between the states is strong. (**b**) When the input vector *u* is the standard Gaussian noise, the coherence between the states is weakened. The thickness degree of the interconnection between nodes represents the strength or the weakness of states coherence.

When there is no input (namely $u=0$), for example, a simpler network with five observational states $({x}_{1},{x}_{2},{x}_{3},{x}_{4},{x}_{5})$, and the states of network nodes have strong coherences in Fig. 2(a). At the same time, the noise leads to a general, one-to-one correspondence between the dynamical coherence and the connections among oscillators for a variety of node dynamics and network structures^21^. In order to reduce the states coherence of the network nodes, we choose the standard Gaussian noise as the input vector *u*, and we expect to achieve the result as that in Fig. 2(b), i.e., the coherences between various states are decreased, so that the reconstruction problem of complex networks whose states are generated by the linear system can be solved. So the linear network system with control input is

3 $$\dot{X}(t)=AX(t)+Bu(t)$$

where *B* is an $N\times M$-dimensional input matrix. This system is controlled using a *M*-dimensional input vector $u(t)={({u}_{1}(t),{u}_{2}(t),\cdots ,{u}_{M}(t))}^{T}$ imposed by the controller, where in general the same signal ${u}_{i}(t)$ may drive multiple nodes. From the derivation process of Eqs (11–16) in the Methods section, we have

4 $$[{X}^{T}\quad {U}^{T}][\begin{array}{c}{\bar{A}}^{T}\\ {\bar{B}}^{T}\end{array}]={Y}^{T}$$

We decompose the state matrix ${X}^{T}$ of the linear network system by QR decomposition (For the detailed explanation of QR decomposition, please see the derivation process of Eqs (20–21) in the Methods section), and we can obtain

5 $${S}_{2}^{T}{U}^{T}{\bar{B}}^{T}={S}_{2}^{T}y$$

6 $$\bar{A}^{T}={R}_{1}^{-1}{S}_{1}^{T}(y-{U}^{T}{\bar{B}}^{T})$$

where ${S}_{2}^{T}{U}^{T}$ is the $(P-N)\times M$-dimensional measurement matrix which is constructed by Gaussian noise, and it should satisfy some conditions such as coherence, RIP, zero space and etc. Then we can reconstruct matrix *B* based on compressed sensing, and reconstruct the network structure *A*. For the detailed explanation of compressed sensing, please see the Methods section. From Eq. (6), we also discover that the noise can build the bridge between the dynamics and the topological structure in order to realize the network reconstruction.

In the presence of control input, the coherences of measurement matrices for six real networks and four model networks are strong, and these networks can not be reconstructed based on compressed sensing. However, the Gaussian noise is linearly independent, which makes it is possible for the measurement matrix ${S}_{2}^{T}{U}^{T}$ to meet the coherence conditions. When the input vector *u* uses the standard Gaussian noise, the coherence changes of the measurement matrices constructed by Eq. (5) in six real networks (Polbooks, Celegansneural, Dolphins, Football, Jazz and ZK) and four model networks (NW, WS, ER and BA) are shown in Fig. 1(b,d) as *nt* (now $nt=(P-N)/M$, $1.2N\le P\le 2N$, and we fix $M=N$) increases. The values of the size *N* for these networks are given in Table 1, and the measurement data are selected from the time $t=350$. In Fig. 1(b,d), the curves with different marks show the coherence changes of different measurement matrices for six real networks and four model networks. From Fig. 1(b), we can see that the coherence curves of these six constructed measurement matrices for six real networks (Polbooks, Celegansneural, Dolphins, Football, Jazz and ZK) jump in the linear network system with the control input, but the overall trend is declining. Compared with Fig. 1(a), it is obvious that the coherence values of these six constructed measurement matrices in Fig. 1(b) are reduced. The coherence values of these six constructed measurement matrices are between 0.3 and 0.6 where $nt\ge 0.3$. Figure 1(d) shows that the coherence curves of these four constructed measurement matrices for the model networks (NW, WS, ER and BA) present a smooth jump, and the overall trend is also declining. When $nt\ge 0.3$, the coherence values of these four measurement matrices are maintained between 0.35 and 0.6. Therefore, the measurement matrix constructed by Gaussian noise has a smaller coherence, the noise drives the states of network nodes oscillate which breaks the states stability of the linear network system, and the network can be reconstructed using the compressed sensing method. In other words, after adding the Gaussian noise as the control input, it is possible to reconstruct matrix *B* by the compressed sensing method, and then we can reconstruct the network structure *A*. Thus, the proper use of noise can bring advantage to the reconstruction of complex networks whose nodes states are generated from the linear network system.

### Factors and success rates of networks reconstruction

**Figure 3:**
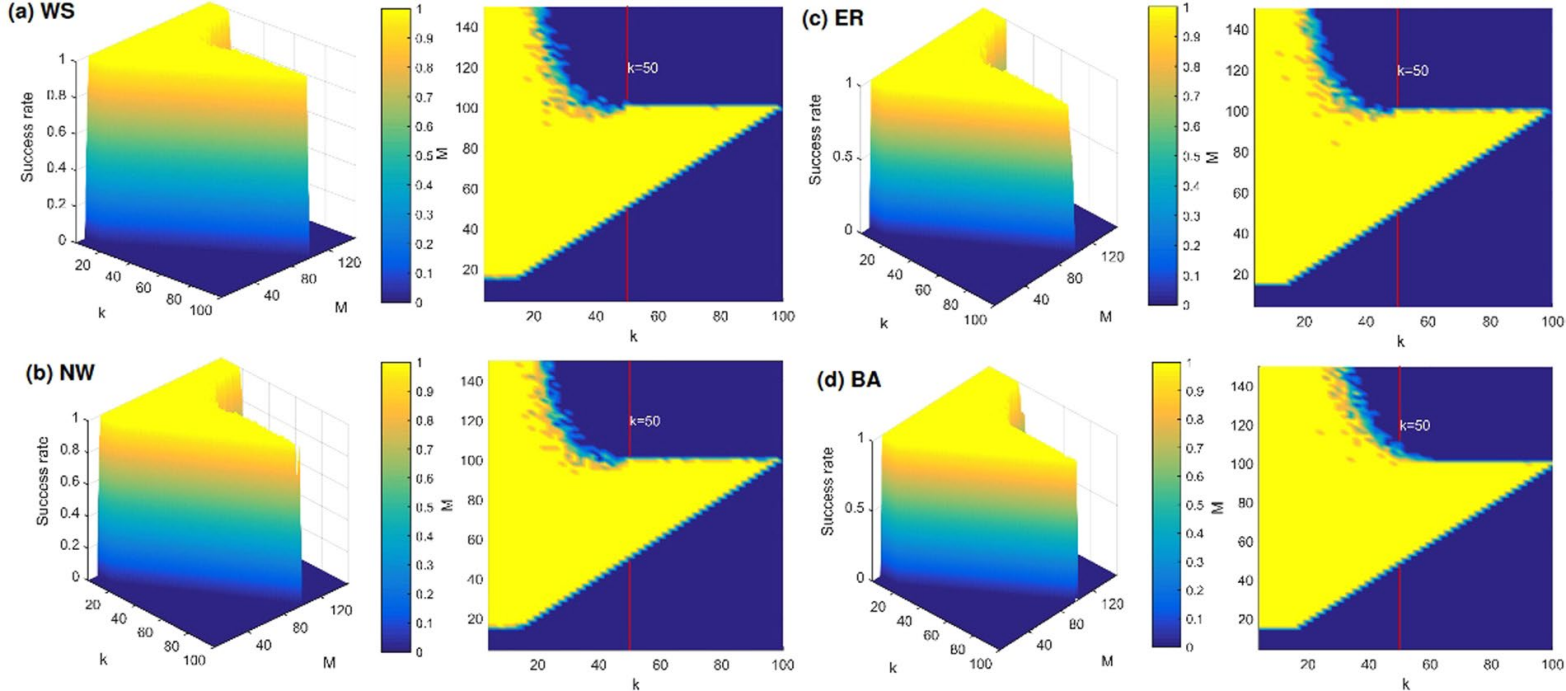
Success rates versus different columns *M* and different sparsities *k* of matrix *B* for WS, NW, ER and BA networks with network size $N=50$. These experiments select measurement data from the time $t=350$, the input vector *u* is the $M\times P$-dimensional standard Gaussian noise, $M=100$ and $P=150$. The success rate is defined as the ratio between the simulation number of successful reconstruction *α* and the simulation number *β*. In these experiments, 20 simulations are performed, and the error of each simulation is $\varepsilon  < {10}^{-6}$.

From the detailed introduction of compressed sensing in the Methods section, we can see that when the sparse signal is reconstructed with compressed sensing, the sparsity of the signal should meet $k\ll M$, and the measurement matrix should meet $(P-N) < M$. Therefore, we study the relationship among the reconstruction success rates of WS, NW, ER and BA networks, the sparsity *k* of the input matrix and the column *M* of matrix *B* in Fig. 3. In the experiments, the sizes *N* of these four model networks are 50, the elements of matrix *B* are randomly selected as 0 or 1, the input vector *u* is the standard Gaussian noise, and we select $M=100$ and $P=150$. When the success rates of these four networks achieve 100%, the graphs in Fig. 3(a–d) appear substantially the same. In Fig. 3, the measurement matrix constructed according to Eq. (5) is an underdetermined matrix when $M > 100$. Based on compressed sensing, these networks can be reconstructed with a small amount of measurement data. When the sparsity is $k\le 50$ and $M > 100$, the complex networks can be reconstructed, and the sparsity of the signal satisfies both $(P-N)\ge 2k$ and $k\ll M$. But the network can not be successfully reconstructed when $k > 50$ and $M > 100$. At this time, the added noise will disturb the state of the system which leads that the network can not be reconstructed. So we should select suitable values of the sparsity for matrix *B* which should be chosen as $1\le k\le 25$ for the network reconstruction. The measurement matrix is an overdetermined matrix when $M\le 100$. The range of the sparsity for matrix *B* is $0 < k\le M$ when the success rate of network reconstruction obtains 100%. Why is it only required that the sparsity of matrix *B* can be selected as a value less than or equal to *M* for networks reconstruction when $M\le 100$? The problem is whether we only should consider the choice of $M\le 100$ to simplify the network reconstruction. We will continue to discuss this important issue in future studies.

**Figure 4:**
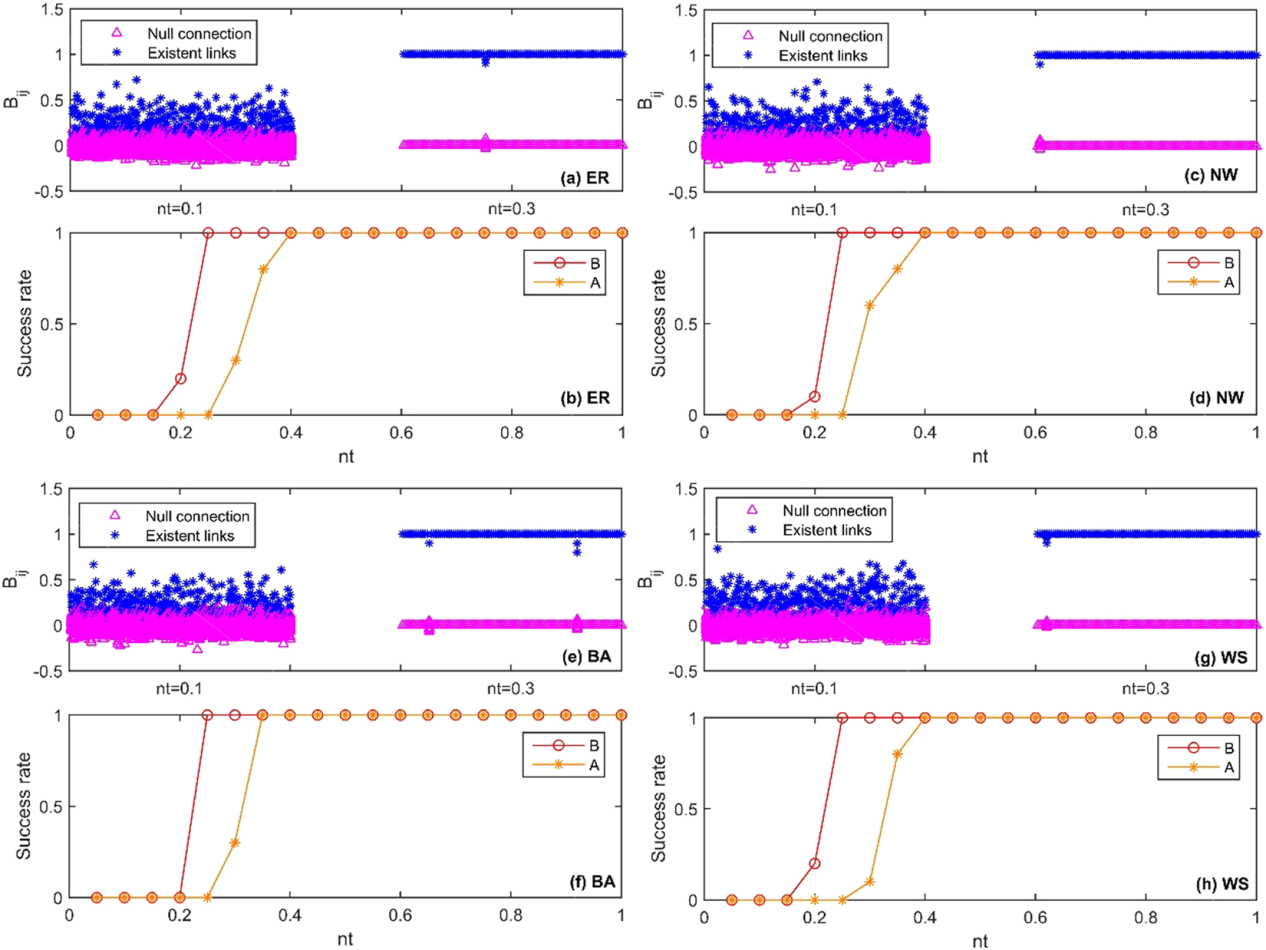
Network reconstruction performances. (**a**,**c**,**e**,**g**) Element values ${B}_{ij}$ vs. different *nt* for the linear network system. (**b**,**d**,**f**,**h**) Relationship between reconstruction success rate and *nt* of network structure matrix *A* and matrix *B* for four model networks (i.e. ER, NW, BA and WS networks). The networks size *N* is 100 with average node sparsity $\langle k\rangle =4$. The elements of matrix *B* are randomly selected as 0 or 1, and the sparsity of controlled matrix *B* is 4 (namely ${\parallel {\bar{B}}^{T}\parallel }_{0}=4$). The input vector *u* is the $M\times P$-dimensional standard Gaussian noise. These experiments select measurement data from the time $t=350$, *nt* is the ratio between the row and the column of the measurement matrix ($nt=(P-N)/M$), *P* is the number of experiments $(N+1\le P\le 3N)$, and $M=200$. The success rate is defined as the ratio between the number of successful simulation $\alpha$ and the simulation number $\beta$. In these experiments, 20 simulations were performed, and the error of each simulation is $\varepsilon  < {10}^{-6}$.

It can be seen from the theoretical derivation process of Eqs (19–23) in the Methods section, the input matrix *B* plays an important role in the process of network reconstruction. Firstly we need to reconstruct the input matrix *B* based on the method of compressed sensing, and then we reconstruct the network *A* according to Eq. (6). Therefore, we should study the effect of the reconstruction of input matrix *B* on the reconstruction of network *A*. In Fig. 4, we adopt the BA, ER, NW and WS networks with average node sparsity $\langle k\rangle =4$, and the sizes of these four networks are 100. The elements of matrix *B* are randomly selected as 0 or 1, the sparsity of controlled matrix *B* is 4 (namely ${\parallel {\bar{B}}^{T}\parallel }_{0}=4$), and the input vector *u* is the $M\times P$-dimensional standard Gaussian noise matrix, where $nt=(P-N)/M$, $N+1\le P\le 3N$, and $M=200$. The measurement data are selected from the time $t=350$. The success rate is defined as the ratio between the simulation number of successful reconstruction $\alpha$ and the simulation number $\beta$. In these experiments, 20 simulations were performed, and the error of each simulation is $\varepsilon  < {10}^{-6}$. We reconstruct matrix *B* at two different values of *nt* in Fig. 4(a,c,e,g), from which we can see that the elements of matrix *B* have overlapping parts at $nt=0.1$, but the elements of matrix *B* have been clearly distinguished at $nt=0.3$. Furthermore, in order to analyze the relationship between the reconstructed matrix *B* and the reconstructed structure *A* of ER, NW, BA and WS networks, we give the curves of reconstruction success rates for matrices *B* and *A* of these four networks (ER, NW, BA and WS networks) with the increment of *nt* in Fig. 4(b,d,f,h). For those matrices *B* that are selected in these four networks in Fig. 4(b,d,f,h), $nt\ge 0.2$ is required for all these four networks so that the success rate of matrix *B* achieves 100%. However, $nt\ge 0.4$ is required for the reconstructions of ER, NW, WS networks and $nt\ge 0.35$ is needed for the reconstruction of BA network so that the reconstruction success rate of network structure *A* can reach 100%. For the minimal measurement data needed to reconstruct matrix *B*, the ER, NW, BA and WS networks can not be reconstructed. For the reconstruction of these four networks, it is required that the system state ${X}^{T}$ should be firstly resolved by QR decomposition, and then these networks can reconstructed according to Eq. (6). But the inverse matrix of ${R}_{1}$ can not be solved when the measurement data are not enough. Therefore, it should use more measurement data to reconstruct ER, NW, BA and WS networks.

### Comparison of two reconstruction methods

In the above sections, we mentioned that we could not reconstruct network structure *A* from Eq. (2) by CS method. In addition, we also try to reconstruct network structure *A* from Eq. (4) by directly using CS method, and find that the success rates of networks reconstruction are all 0. However, if the state matrix *X* is replaced by the stochastic Gaussian matrix, we show that network structure *A* can be solved in a certain amount of measurement data by CS method. Here, we compare the QR-CS method with the CS method (where state matrix *X* is replaced by the stochastic Gaussian matrix) to observe the effects of these two methods on the reconstruction of complex networks.

**Figure 5:**
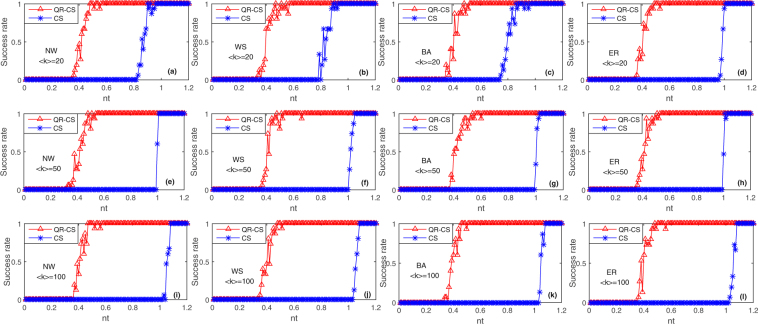
Reconstruction success rates as a fraction of *nt* for NW, WS, BA and ER networks to compare two methods. The networks size *N* is 100. The elements of matrix *B* are randomly selected as 0 or 1, and the sparsity of the controlled matrix *B* is 4 (namely ${\parallel {\bar{B}}^{T}\parallel }_{0}=4$). The input vector *u* is the $M\times P$-dimensional standard Gaussian noise. These experiments select measurement data from the time $t=350$, *nt* is the ratio between the row and the column of the measurement matrix (in the QR-CS method, $nt=(P-N)/M$, $N+1\le P\le 2.2N$, and in the CS method (where state matrix *X* is replaced by stochastic Gaussian matrix), $nt=P/N$, $1\le P\le 1.2N$), and $M=100$. The success rate is defined as the ratio between the simulation number of successful reconstruction $\alpha$ and the simulation number $\beta$. In these experiments, 20 simulations are performed, and the error of each simulation is $\varepsilon  < {10}^{-6}$. (**a**–**d**) For these four model networks, the average node sparsity is $\langle k\rangle =20$. (**e**–**h**) $\langle k\rangle =50$. (**i**–**l**) $\langle k\rangle =100$.

The proposed method can reconstruct not only the sparse networks but also the dense networks. We compare the reconstruction success rates of the QR-CS method with those of the CS method for the reconstruction of NW, WS, BA, and ER networks in Fig. 5. We select different three average sparsities (i.e. $\langle k\rangle =20$, $\langle k\rangle =50$, $\langle k\rangle =100$) for these four model networks to simulate, and $M=100$, $N=100$. In the QR-CS method, $nt=(P-N)/M$ $(N+1\le P\le 2.2N)$, but in the CS method (where state matrix *X* is replaced by the stochastic Gaussian matrix), $nt=P/N$ $(1\le P\le 1.2N)$. And other parameters are the same as those in Fig. 4. The average node sparsities of these four model networks are 20 in Fig. 5(a–d), from which we can see that the reconstruction success rates of these four model networks with QR-CS method achieve 100% when $nt\ge 0.57$, and they present stable trends. However, the CS method requires larger *nt* to achieve 100% success rates of these networks, compared with the QR-CS method. When *nt* approaches 1, the reconstruction success rate of the CS method increases. The average node sparsities of these four model networks are 50 in Fig. 5(e–h). We can see from Fig. 5(e–h) that the success rates of these four networks reconstructed by QR-CS method all reach 100% when $nt\ge 0.61$, and they are in the stable trends. While using the CS method, it needs $nt > 1$ to reconstruct these networks. We select the dense NW, WS, BA and ER networks to compare these two methods in Fig. 5(i–l), where $\langle k\rangle =100$. It can be seen from Fig. 5(i–l) that based on QR-CS method dense networks only require $nt\ge 0.5$ to make the success rates of networks reconstruction reach 100%, but using CS method we still needs $nt > 1$ to reach 100% success rates of networks reconstruction. The random initial state vector is selected in each experiment, so at the initial period the curves of the success rates of network reconstruction present the jumps. In these three groups of experiments with three different sparsities in Fig. 5, the success rates of networks reconstruction by QR-CS method are basically stable when $nt > 0.61$. The comparison results for these three groups of experiments in Fig. 5 show that the QR-CS reconstruction method is better than the CS reconstruction method.

**Figure 6:**
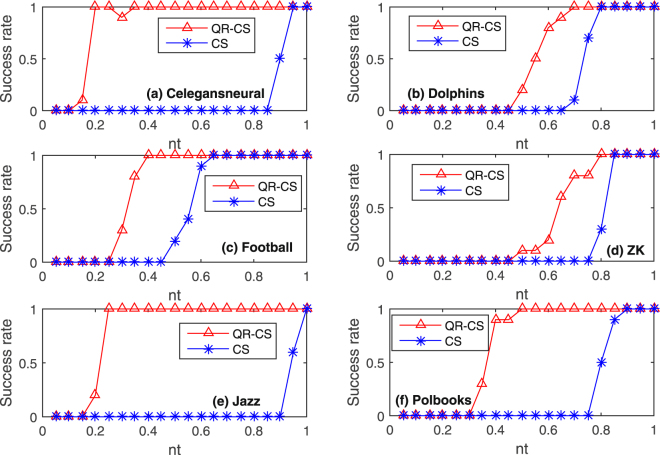
Comparisons between these two methods (i.e. QR-CS and CS methods) on the reconstruction success rates as function *nt* changes in Celegansneural, Dolphin, Football, ZK, Jazz and Polbooks networks. The values of networks size *N* and their average sparsities are shown in Table 1. These experiments select the measurement data from the time $t=350$, *nt* is the ratio between the row and the column of the measurement matrix (in the QR-CS method, $nt=(P-N)/M$, $N+1\le P\le 2N$, and in the CS method (where state matrix *X* is replaced by stochastic Gaussian matrix), $nt=P/N$, $1\le P\le N$), and $M=N$. The input vector *u* is the $M\times P$-dimensional standard Gaussian noise. The success rate is defined as the ratio between the simulation number of successful reconstruction $\alpha$ and the simulation number $\beta$. In these experiments, 20 simulations are performed, and the error of each simulation is $\varepsilon  < {10}^{-6}$.

In order to further verify the performance of QR-CS method described in this paper for the reconstruction of sparse networks, we give the curves of reconstruction success rates for six real networks (namely, Celegansneural, Dolphins, Football, Jazz, ZK and Polbooks) by QR-CS method and CS method with the increment of *nt* in Fig. 6. In the experiment, we choose $M=N$, where *N* is the size of the network. The values of the size *N* and the average sparsities of the networks are shown in Table 1, and other parameters are selected the same as those in Fig. 5. In this paper, when reconstructing the networks with the proposed QR-CS method, different sizes of the networks require different *nt* so that the success rates of networks reconstruction can reach 100%. For the Celegansneural and Jazz networks whose sizes are larger, *nt* is required to be about 0.25 so that the reconstruction success rates can achieve 100%, but for the Dolphin and ZK networks whose sizes are smaller, *nt* is required to be about 0.8 so that the reconstruction success rates can achieve 100%. For Football and Polbooks networks, when *nt* is about 0.4, the reconstruction success rates can reach 100%. Compare with QR-CS method, relatively larger *nt* is required for networks reconstruction by CS method. That is, compare with CS method, less measurement data are required by the proposed QR-CS method to construct the networks after adding the noise as the control input. In Fig. 6, the success rates of the reconstruction present the transitions, because the initial random state vector is chosen in each experiment.

In a word, it is clear that the QR-CS method proposed in this paper is more efficient than the CS method by the comparisons of experimental results for the reconstructions of four model models and six real networks.

## Conclusions and Discussion

In summary, we proposed a reconstruction method of complex networks from measurable time series. Different with the existing methods that considered enough data were needed to achieve the reconstruction and the noise often harmed the network reconstruction, we discovered that less measurement data were required by the proposed QR-CS method to reconstruct the network after adding the Gaussian noise, which would increase the success rate of network reconstruction.

By the discretization of the continuous variable, the model of complex networks generated by the linear system was transformed into a mathematical form that could be solved by the theory of compressed sensing. We discovered that without the control input, the method of compressed sensing could not succeed in reconstructing such complex networks in which the states of nodes were generated through the linear network system. We took the simulation results for the reconstruction of six different real networks and four model networks as examples and analyzed the reason why these networks without the control input could not be reconstructed by only compressed sensing. The state of simple linear systems is stable and it has a strong correlation, which is unfavourable to the reconstruction of complex networks generated by the linear system. However, the noise can drive the dynamics between nodes to break the stability of the system state. In order to decrease the coherence of the system states, the noise was introduced as the control input and it was beneficial for the reconstruction of such complex networks whose node states were generated by the linear network system. We presented the curves of coherence changes for four model networks and six real networks with Gaussian noise as the input to verify that the noise could decrease the coherence of networks states generated by the linear network system. A new method integrating QR decomposition and compressed sensing was proposed to solve the reconstruction problem of complex networks under the assistance of the input noise. The state matrix of the system was decomposed by QR decomposition. And we constructed the measurement matrix with the aid of Gaussian noise so that the sparse input matrix could be reconstructed by compressed sensing. Furthermore, the structure of complex network could be reconstructed. Experiments were presented to show that the proposed method was more accurate and more efficient to reconstruct four model networks and six real networks by the comparisons between the proposed method and only compressed sensing. We found that the input matrix was vital to the reconstruction process of complex networks, and we studied the influences of input matrix on the reconstruction of networks. The proposed method is still more accurate and more efficient to reconstruct the dense networks. We compared the success rates of the proposed QR-CS method with those of CS method for the reconstruction for four model networks with different sparsities. Although some problems remain in this article, it is important for us to further study complex networks reconstruction in a linear network system.

There are many sparse networks in complex networks, and there are many dense networks correspondingly. Complex networks reconstructed based on compressed sensing should be sparse in the existing studies, which has some limitations on the reconstruction of dense complex networks. If the dense network is reconstructed by compressed sensing, some measures must be taken to make it sparse. Some errors can occur in this process, which leads to erroneous network reconstruction results. However, these important aspects have received little attention in the existing studies. This paper breaks through the limitations of using compressed sensing to reconstruct sparse networks with smaller amount of measurement data. The proposed method can reconstruct not only sparse complex networks, but also dense complex networks. In addition, we do not need to take measures to make complex networks sparse. Obviously, our method provides a new way to solve the reconstruction of dense complex networks which can be used to infer the dense subgraphs from the gene expression data in the biological network or to infer the layout of the dense traffic network, and so on. Further, the relationship networks between people become unprecedented dense by Facebook, Twitter and other social networking site, and our proposed method can be used to better analyze the interpersonal relationships. At the same time, noise is also an unavoidable factor in the reconstruction of complex networks. Previous works suggested that noise had influences on the reconstruction of complex networks, which could disturb the measurement data or reduce the success rate of networks reconstruction. However, we found that the linear network system that introduced noise was capable of reconstructing complex networks, and the proposed method has a high success rate. It shows from a side view that noise can build the bridge between the dynamics and the topological structure in order to realize complex networks reconstruction.

## Methods

### Compressed sensing

As a theory of signal processing, compressed sensing was firstly proposed by Donoho *et al*.^34^. Once it was put forward, compressed sensing has received highly concerns in many fields, such as information theory, image processing, network control, computer science, and wireless communication^35^. The main idea of compressed sensing is to observe and compress an *N*-dimensional signal $\eta$ (if this signal is sparse or can be compressed), and thus obtain an *M*-dimensional observational value *y*, whose main form is

7 $${\rm{\Phi }}\eta =y$$

where ${\rm{\Phi }}$ is an $M\times N$-dimensional measurement matrix and $M < N$. From the theory of linear equations, we can know that the above mentioned Eq. (7) is an ill-conditioned equation or an underdetermined equation. If the signal $\eta$ is *k*-sparse, $k\ll N$, and the measurement matrix satisfies some conditions such as coherence^35^, RIP^36^, zero space^37^ and etc., then the signal $\eta$ can be recovered from the observational value *y*.

The coherence of measurement matrix ${\rm{\Phi }}$, $\mu ({\rm{\Phi }})$, is the largest absolute inner product between any two columns ${{\rm{\Phi }}}_{i}$, ${{\rm{\Phi }}}_{j}$ of ${\rm{\Phi }}$

8 $$\mu ({\rm{\Phi }})=\mathop{{\rm{\max }}}\limits_{1\le i,j\le N}{\frac{| \langle {{\rm{\Phi }}}_{i},{{\rm{\Phi }}}_{j}\rangle | }{{\parallel {{\rm{\Phi }}}_{i}\parallel }_{2}\parallel {{\rm{\Phi }}}_{j}\parallel }}_{2}$$

The smaller the coherence of the measurement matrix is, the higher the accuracy of the reconstructed signal $\eta$ achieves. When the measurement matrix satisfies the coherence condition, since the signal $\eta$ is sparse, Equation (7) can be solved by ${l}_{0}$-norm

9 $$\,{\rm{\min }}\,{\parallel \eta \parallel }_{0}\quad \,{\rm{subject}}\,{\rm{to}}\quad {\rm{\Phi }}\eta =y$$

However, finding the solution of ${l}_{0}$-norm is an NP-hard problem. So we use ${l}_{1}$-norm optimization to approximately solve the problem

10 $$\,{\rm{\min }}\,{\parallel \eta \parallel }_{1}\quad {\rm{subject}}\,{\rm{to}}\quad {\rm{\Phi }}\eta =y$$

Then, Equation (7) can be solved by OMP^38^, BP^39^ and other algorithms. Our goal is to reconstruct complex networks in this paper. We transform the problem of network reconstruction into the solution problem of underdetermined equations. This underdetermined equation can be solved by the ${l}_{1}$-norm, and then solved by the OMP algorithm. Thus, we can reconstruct complex networks from the observational matrix and the measurement matrix.

### Linear network system model

We consider the following linear network system

11 $$\dot{X}(t)=AX(t)+Bu(t)$$

where the vector $X(t)={({x}_{1}(t),{x}_{2}(t),\cdots ,{x}_{N}(t))}^{T}$ is the state of *N* nodes for a network system at time *t*, and the $N\times N$-dimensional matrix *A* is the network structure between nodes. The gene regulation network^10,11^, the propagation network^12^ and etc. can be written in the form of Eq. (11). In this linear network system, *B* is an $N\times M$-dimensional input matrix. And the system is controlled using a *M*-dimensional input vector $u(t)={({u}_{1}(t),{u}_{2}(t),\cdots ,{u}_{M}(t))}^{T}$ imposed by the controller, where in general the same signal ${u}_{i}(t)$ may drive multiple nodes.

For $\dot{x}_{i}(t)$, we have

12 $$\dot{x}_{i}(t)=\sum _{j=1}^{N}{a}_{ij}{x}_{j}(t)+\sum _{k=1}^{M}{b}_{ik}{u}_{k}(t)$$

where ${a}_{ij}$ represents the element of matrix *A*, ${b}_{ik}$ represents the element of matrix *B*. If the continuous system (12) is computed on a digital computer, it must be discretized^40^. Similar with the existing works^40,41^ about discretization, we have the following formula

13 $$\dot{x}_{i}({t}_{q})=\frac{{x}_{i}({t}_{q+1})-{x}_{i}({t}_{q})}{{\rm{\Delta }}t}$$

where ${\rm{\Delta }}t={t}_{q+1}-{t}_{q}\ll 1$, and *q* is an integer. Then, we have

14 $${x}_{i}({t}_{q+1})=\sum _{j=1}^{N}({\rm{\Delta }}t{a}_{ij}+{e}_{ij}){x}_{j}({t}_{q})+\sum _{k=1}^{M}{\rm{\Delta }}t{b}_{ik}{u}_{k}({t}_{q})$$

where $${e}_{ij}=\{\begin{array}{cc}1 & i=j\\ 0 & i\ne j\end{array}$$.

The system is represented compactly in the matrix form as follows

15 $${Y}^{(i)}=\bar{A}{X}^{(i)}+\bar{B}{U}^{(i)}$$

where ${Y}^{(i)}={X}_{i}({t}_{q+1})$, ${X}^{(i)}={X}_{i}({t}_{q})$, $\bar{A}:={\rm{\Delta }}tA+E$, $\bar{B}:={\rm{\Delta }}tB$, and ${U}^{(i)}={U}_{i}({t}_{q})$. ${U}^{(i)}$ and ${Y}^{(i)}$ represent the input and output vectors and ${X}^{(i)}$ is the state vector in the *i* th experiment. It is assumed that at each time a different vector ${U}^{(i)}$ is selected for *P* experiments, but the rest vectors of ${X}^{(i)}$ for the rest experiments are generated by the system after selecting the vector ${X}^{(i)}$ in the first experiment. The input matrix, the output matrix and the state matrix for these *P* experiments are given as follows

$$\begin{array}{lll}U & := & [\begin{array}{cccc}{U}^{(1)} & {U}^{(2)} & \cdots \, & {U}^{(P)}\end{array}]\\ Y & := & [\begin{array}{cccc}{Y}^{(1)} & {Y}^{(2)} & \cdots \, & {Y}^{(P)}\end{array}]\\ X & := & [\begin{array}{cccc}{X}^{(1)} & {X}^{(2)} & \cdots \, & {X}^{(P)}\end{array}]\end{array}$$

So $Y=\bar{A}X+\bar{B}U$ can be written as

16 $$[\begin{array}{cc}{X}^{T} & {U}^{T}\end{array}][\begin{array}{c}{\bar{A}}^{T}\\ {\bar{B}}^{T}\end{array}]={Y}^{T}$$

where $$[\begin{array}{cc}{X}^{T} & {U}^{T}\end{array}]$$ is a $P\times (N+M)$-dimensional matrix. Thus, we convert the linear system model into the form of Eq. (7), and assume that the dynamical structure $(\bar{A},\bar{B})$ can be estimated from Eq. (16) by the method of compressed sensing.

### Network reconstruction without control input

For the linear network system model, when there is no external input, that is, the input vector $u=0$, the linear network system is

17 $$Y=\bar{A}X$$

Then, Equation (17) can be written as

18 $${Y}^{T}={X}^{T}{\bar{A}}^{T}$$

Here, we consider the reconstruction problem of six different real networks, namely, Polbooks^12^, Celegansneural^25^, Dolphins^26^, Football^27^, Jazz^28^, and ZK^29^ without control input. We also investigate on the reconstruction of four model networks, i.e. Newman-Watts small-world network (NW)^30^, Watts-Strogatz small-world network (WS)^31^, Erdos-Renyi random network (ER)^32^, and Barabasi-Albert scale-free network (BA)^33^ without control input. Numerical simulation results of Table 1, Fig. 1(a,c), and the corresponding detailed analyses in the Results section show that the networks whose node states are generated by the linear network system can not be reconstructed only by the method of compressed sensing when the input vector $u=0$.

### Network reconstruction with control input

A dynamical system is controllable if it has suitable inputs, then its states can be driven from any initial states to any desired final states within a finite time^42^. At the same time, the noise leads to a general, one-to-one correspondence between the dynamical coherence and the connections among oscillators for a variety of node dynamics and network structures^21^. Therefore, we can add the noise to change the status of nodes in the linear networks system. There is a strong coherence between the states of network nodes without input in Fig. 2(a) (please see the Results section), and the coherence between the states of network nodes is reduced by adding the noise as the input in Fig. 2(b).

When the input vector $u\ne 0$, Equation (16) and Equation (7) are similar in the mathematical form, so Equation (16) can be written as

19 $${\rm{\Phi }}\eta =y$$

where $${\rm{\Phi }}\,:=[\begin{array}{cc}{X}^{T} & {U}^{T}\end{array}]\in {R}^{P\times (N+M)}$$, $$\eta :=[\begin{array}{c}{\bar{A}}^{T}\\ {\bar{B}}^{T}\end{array}]\in {R}^{(N+M)\times N}$$, and $y:={Y}^{T}\in {R}^{P\times N}$. Here, ${\rm{\Phi }}$ and *y* can be measured by time series. So we choose the method of compressed sensing to reconstruct the matrix $\eta$. The input matrix *B* can be controlled, assuming ${\parallel {\bar{B}}^{T}\parallel }_{0}=k$ ($k\ll M$), but ${\parallel {\bar{A}}^{T}\parallel }_{0}$ is unknown. It is necessary to know the sparsity of the matrix to reconstruct the network by compressed sensing^43–45^. Here, we can not directly apply the compressed sensing into Eq. (19) to reconstruct the network structure *A*. So we should use the following QR method.

QR decomposition can decompose matrix ${X}^{T}$ into the product of an orthogonal matrix and an upper triangular matrix, so that we can make full use of the sparse property of matrix *B* and the compressed sensing method to solve matrix *B*. Take the QR decomposition of ${X}^{T}\in {R}^{P\times N}$, we get

20 $${X}^{T}=[\begin{array}{cc}{S}_{1} & {S}_{2}\end{array}][\begin{array}{c}{R}_{1}\\ 0\end{array}]$$

where $$[\begin{array}{cc}{S}_{1} & {S}_{2}\end{array}]\in {R}^{P\times P}$$ is an orthogonal matrix and ${R}_{1}\in {R}^{N\times N}$ is an upper triangular matrix.

Pre-multiply Eq. (16) by $${[\begin{array}{cc}{S}_{1} & {S}_{2}\end{array}]}^{T}$$, we have

21 $$[\begin{array}{cc}{R}_{1} & {{S}_{1}}^{T}{U}^{T}\\ 0 & {{S}_{2}}^{T}{U}^{T}\end{array}]\,[\begin{array}{c}{\bar{A}}^{T}\\ {\bar{B}}^{T}\end{array}]=[\begin{array}{c}{{S}_{1}}^{T}y\\ {{S}_{2}}^{T}y\end{array}]$$

According to the multiplication of the second row of the first matrix in Eq. (21) and the second matrix in Eq. (21), we can get

22 $${S}_{2}^{T}{U}^{T}{\bar{B}}^{T}={S}_{2}^{T}y$$

where ${S}_{2}^{T}{U}^{T}\in {R}^{(P-N)\times M}$ ($P-N\ll M$), and ${\parallel {\bar{B}}^{T}\parallel }_{0}=k$ ($k\ll M$). We solve ${\bar{B}}^{T}$ according to Eq. (22). It is only required to make sure that the measurement matrix ${S}_{2}^{T}{U}^{T}$ in Eq. (22) satisfies some conditions such as coherence, RIP, zero space and etc., then we can accurately reconstruct matrix ${\bar{B}}^{T}$ by the reconstruction algorithm of compressed sensing. Meanwhile, Candès *et al*.^36^ pointed out that if $P-N\ge 2k$ and all the subsets of the $2k$ columns of the measurement matrix were linearly independent, then the *k*-sparse signal could be reconstructed by compressed sensing. Gaussian noise is linearly independent^46^, which makes it is possible for the measurement matrix ${S}_{2}^{T}{U}^{T}$ to meet the coherence condition. When the vector *u* uses the standard Gaussian noise, the coherence changes of the measurement matrices constructed by Eq. (22) in six networks (Polbooks, Celegansneural, Dolphins, Football, Jazz and ZK) are studied by the proposed QR-CS method. We also investigate on the reconstruction of four model networks, i.e. Newman-Watts small-world network (NW)^30^, Watts-Strogatz small-world network (WS)^31^, Erdos-Renyi random network (ER)^32^, and Barabasi-Albert scale-free network (BA)^33^ when the control input is the standard Gaussian noise. From the experimental results of Fig. 1(b,d) and the corresponding detailed analyses in the Results section, we find that it is possible to reconstruct matrix *B* by the reconstruction algorithm of compressed sensing.

If ${R}_{1}$ is full rank which requires ${X}^{T}$ to be full column rank, we can solve the network ${\bar{A}}^{T}$ according to the multiplication of the first row of the first matrix in Eq. (21) and the second matrix in Eq. (21), we can get

23 $${\bar{A}}^{T}={R}_{1}^{-1}{S}_{1}^{T}(y-{U}^{T}{\bar{B}}^{T})$$

From Equation (23), we understand that the noise can build the bridge between the dynamics and the topological structure in order to realize the network reconstruction. In the derivation process of Eqs (19–23), not only the reconstruction algorithm of compressed sensing but also the QR decomposition are used. With the assistance of the noise, the network can be reconstructed accurately from the measurable time series. From the derivation process of Eqs (19–23), it can be concluded that the network is not necessarily required to be sparse when reconstructing the network, and the dense networks also can be reconstructed.

In summary, we have given a general method to solve the problem of complex networks generated through a linear network system. However, besides the linear continuous system, for a linear discrete system $x(t+{\rm{1)}}=Ax(t)+Bu(t)$, we can also consider using the proposed method to realize the reconstruction of complex networks.

### Reconstruction algorithm

The reconstruction algorithm based solely on compressed sensing (CS method) can not solve the problem of network reconstruction in linear network systems. So we propose the QR-CS reconstruction algorithm to solve the network reconstruction in linear network systems according to Eqs (19–23). The pseudocode of this QR-CS algorithm is given in Table 2. The process of QR-CS algorithm is given as follows. Input matrices *U* and *B*, set the sparsity of matrix *B* in the first step, and calculate *X* and *Y* in Steps 2–5. Then *X* is resolved by QR decomposition in Step 6, and reconstruct *B* based on the reconstruction algorithm of compressed sensing in Steps 7–9. Finally recover *A* in Steps 10–12. This algorithm is terminated.

**Table 2:** Pseudocode of the QR-CS reconstruction algorithm.

Algorithm 1 QR-CS reconstruction algorithm
Require:$$Y\in {R}^{N\times P}$$, $$X\in {R}^{N\times P}$$ and $$U\in {R}^{M\times P}$$
Ensure: $$A\in {R}^{N\times N}$$ and $$B\in {R}^{N\times M}$$
1: input $$U\in {R}^{M\times P}$$, set $${\parallel B\parallel }_{0}=k$$
2: for $i=1$ to $P+1$ do
3: $${X}^{i}={({x}_{1},{x}_{2},\cdots ,{x}_{N})}^{T}.$$
4: $${Y}^{i}={({y}_{1},{y}_{2},\cdots ,{y}_{N})}^{T}.$$
5: end for
6: $$X\in {R}^{N\times P}\mathop{\to }\limits^{QR\,decomposition}[{S}_{1},{S}_{2}][\begin{array}{c}{R}_{1}\\ 0\end{array}]$$
7: for $i=1$ to $N$ do
8: $${B}^{i}\mathop{\leftarrow }\limits^{CS\,reconstruction\,algorithm}({{{\rm{S}}}_{2}}^{T}* {Y}^{i},{{{\rm{S}}}_{2}}^{T}{* U}^{T}).$$
9: end for
10: $${B}_{i}\to {A}_{i}$$
11: $$A=[{A}^{1},{A}^{2},\cdots ,{A}^{N}]\in {R}^{N}.$$
12: return *A*.
